# Supplementary material for: Persistent and multiclonal malaria parasite dynamics despite extended artemether-lumefantrine treatment in children
Source: Nat Commun. 2024 May 7;15:3817. doi: 10.1038/s41467-024-48210-7 (PMC11076639; doi:10.1038/s41467-024-48210-7)
Supplement: Supplementary file 1 — Supplementary Information [file 41467_2024_48210_MOESM1_ESM.pdf]

Supplemental Data

Table S1. Molecular studies assessing residual or persistent post-ACT parasitemia

Study (Year) [Reference]	Location	Year	Study Design	Markers (Procedure)	Findings
Beshir et al (2013) [[1]]	Kenya	2009	Randomized, open-label trial of AL or DP for uncomplicated malaria in children aged 0.5-10 years	Pgmet DNA (qPCR), Pfs25 mRNA (QT-NASBA)	Residual day 3 parasitemia was detected by qPCR in 33.3% (28/84) of children treated with AL, and 30.0% (21/70) of children treated with DP. Residual parasitemia was not associated with gametocytemia but was significantly associated with recrudescent and new infections.
Chang et al (2016) [[2]]	Uganda	2011-2013	Randomized, double-blind, placebo-controlled trial of AL alone or AL + 0.75 mg/kg PQ for uncomplicated malaria in children aged 1-10 years	SBP1 mRNA (RT-PCR), Pfs25 mRNA (QT-NASBA)	Persistent ring-stage transcripts were detected at > 25% prevalence until day 14 in both treatment arms. In 88.2% (16/18) of patients, *msp2* genotypes detected on day 14 were also detected on the preceding days.
Tadesse et al (2017) [[3]]	Burkina Faso	2013-2014	Randomized, double-blind, placebo-controlled trial of AL alone or AL + 0.25 or 0.40 mg/kg PQ for asymptomatic malaria in children aged 2-15 years	18S rRNA & rDNA (RT-PCR & qPCR), SBP1, REX1, & PHISTb mRNA (RT-PCR), Pfs25 mRNA (RT-PCR)	Persistent 18S transcripts were detected (63-98% prevalence) until day 14 in all three treatment arms. SBP1 transcripts suggesting ring-stage parasitemia were detected (21-47% prevalence) until day 14 in all three treatment arms. 34.5% (132/383) of day 3+ samples negative for gametocytes were positive for one or more ring-stage markers.
Oyebola et al (2018) [[4]]	Nigeria	2016	Observational study of AL for uncomplicated malaria in patients aged 1-70 years	varATS DNA (qPCR), molecular SNP barcoding	Residual parasitemia detected in 18.5% (12/65) patients on day 3, 21.5% (14/65) on day 7, and 12.3% (8/65) patients on day 28. Molecular barcoding showed high genetic relatedness between day 0 and day 28 parasites in 37.5% (3/8) of patients.
Roth et al (2018) [[5]]	Kenya	2015-2017	Randomized, open-label trial of AL or PA for uncomplicated malaria in children aged 0.5-12 years	18S rDNA (qPCR & db-PCR-NALFIA), Pfs25 mRNA (QT-NASBA)	Residual day 7 parasitemia was detected by qPCR in 37.1% of children treated with AL, and in 46.1% of children treated with PA; day 7 parasitemia by db-PCR-NALFIA was 33.3% with AL, and 30.3% with PA. 27.9% (17/61) of day 7 qPCR positive patients were gametocyte positive.
Mwaiswelo et al (2019) [[6]]	Tanzania	2006-2014	Retrospective study of AL or AL + PQ for uncomplicated malaria in patients aged 0.5-10 years	Cytochrome B (nPCR)	The prevalence of qPCR determined day 3 parasitemia was between 28-74% depending on the study year; this was not associated with microscopic recurrence or recrudescence.
Lubis et al (2020) [[7]]	Indonesia	2015	Randomized, open-label trial of AL or DP (with 0.75 mg/kg PQ) for uncomplicated malaria in patients aged > 0.5 years	Pgmet DNA (qPCR), 18S rDNA (nPCR)	Residual day 3 parasitemia was detected by qPCR in 12% (3/24) and 11% (4/36) of patients treated with AL or DP, respectively. There was evidence of submicroscopic persistent parasitemia on day 28 and day 42 in 36% (20/55) and 41% (24/59) of AL and DP treated groups, respectively.

Mahamar et al (2021) [8]	Mali	2016	Randomized, single-blind trial of SP-AQ, SP-AQ + PQ, DP, or DP + MB, for asymptomatic, gametocyte-positive malaria in patients aged 5-50 years	SBP1 mRNA (RT-PCR), PfMGET & Pfs25 mRNA (RT-PCR)	Persistent ring-stage parasitemia was detected in 37.0% (10/27) and 36.8% (7/19) of DP treated patients on days 7 and 14, respectively. A similar prevalence of ring-stage parasites was detected after SP-AQ treatment (in 36% (9/25) and 36.8% (7/19) of patients on days 7 and 14, respectively).
Beshir et al (2021) [9]	West Africa†	2011-2016	Randomized, multicenter, open-label trial of PA, DP, AS-AQ, or AL in patients aged > 0.5 years	Pgmet DNA (qPCR)	Residual day 3 parasitemia was detected by qPCR in 17.7% (98/552) of children after ACT treatment. Residual day 3 parasitemia after AL treatment was associated with microscopic recurrence. No association was found between day 7 gametocytemia and day 3 qPCR positivity.
Andagalu et al (2022) [10]	Kenya	2015	Longitudinal study using AL for asymptomatic, gametocyte-positive malaria in patients aged ≥ 1 year	18S rRNA & rDNA (RT-PCR), Pfs16 & Pfs25 mRNA (RT-PCR)	Of 373 patients that were positive for asexual parasitemia, 39.4% (147), 33.5% (125), and 5.1% (19) had 1, 2, or 3 episodes of treatment failure, defined as PCR-positive parasitemia one month after treatment, respectively. Treatment failures were associated with younger age and higher transmission periods.
Tadele et al (2022) [11]	Ethiopia	2020	Prospective therapeutic efficacy study of AL for uncomplicated malaria in patients aged > 2 years	varATS DNA (qPCR)	Persistent day 3 and day 7 parasitemia was detected by qPCR in 60% (30/50) and 28% (14/50) of patients, respectively. Seven patients had persistent submicroscopic parasitemia throughout 28 days of follow-up. Day 3 parasitemia was associated with younger age (< 15 years).

AL = Artemether-lumefantrine, DP = Dihydroartemisinin-piperaquine, PQ = Primaquine, PA = Pyronaridine-artesunate, SP-AQ = Sulfadoxine-pyrimethamine/Amodiaquine, MB = Methylene Blue, AS-AQ = Artesunate-Amodiaquine; qPCR = Quantitative Polymerase Chain Reaction, RT-PCR = Quantitative Reverse-transcription PCR, QT-NASBA = Quantitative Nucleic Acid Based Amplification, db-PCR-NALFIA = direct-on-blood PCR Nucleic Acid Lateral Flow Immunoassay, nPCR = Nested PCR.

†Multi-site study that includes Burkina Faso, Gambia, Guinea, and Mali.

Table S2. Study Participant Characteristics

	Treatment group		
	3-Day AL (N = 153)	***5-Day AL (N = 150)***	***Total (N = 303)***
**HIV status**			
Uninfected	114 (74.5%)	113 (75.3%)	227 (74.9%)
Infected	39 (25.5%)	37 (24.7%)	76 (25.1%)
**Episode**			
First	111 (72.5%)	111 (74.0%)	222 (73.3%)
Recurrent	42 (27.5%)	39 (26.0%)	81 (26.7%)
**Sex**			
Male	65 (42.5%)	75 (50.0%)	140 (46.2%)
Female	88 (57.5%)	75 (50.0%)	163 (53.8%)
**Age, y**			
Mean (SD)	7.2 (3.7)	7.4 (3.5)	7.3 (3.6)
**Height, cm**			
Mean (SD)	115.6 (26.5)	116.3 (23.9)	115.9 (25.2)
**Weight, kg**			
Mean (SD)	22.3 (9.2)	21.9 (7.5)	22.1 (8.4)
**Baseline microscopic parasite density (parasites/µL)**			
Geometric mean (95% CI)	7801 (5628, 10813)	7802 (5557, 10955)	7802 (6174, 9859)
**Baseline parasite density, $\log_{10}$(parasites/mL)**			
Microscopic Mean (SD)	6.9 (0.9)	6.9 (0.9)	6.9 (0.9)
18S Mean (SD)	6.3 (0.9)	6.3 (0.9)	6.3 (0.9)
SBP1 Mean (SD)	6.7 (1.0)	6.6 (1.0)	6.7 (1.0)
**Baseline multiplicity of infection**			
Median (IQR)	5.0 (3.3 – 7.0)	4.0 (2.7 – 7.0)	4.5 (3.0 – 7.0)
**WHO outcome, day 28**			
ACPR	76 (52.8%)	91 (62.8%)	167 (57.8%)
LCF	16 (11.1%)	11 (7.6%)	27 (9.3%)
LPF	52 (36.1%)	43 (29.7%)	95 (32.9%)
Missing	9	5	14
**WHO outcome, day 42**			
ACPR	38 (26.2%)	48 (32.9%)	86 (29.6%)
LCF	34 (23.4%)	33 (22.6%)	67 (23.0%)
LPF	73 (50.3%)	65 (44.5%)	138 (47.4%)
Missing	8	4	12
**Recurrent parasitemia, day 28†**			
No	84 (54.9%)	96 (64.0%)	180 (59.4%)
Yes	69 (45.1%)	54 (36.0%)	123 (40.6%)
**Recurrent parasitemia, day 42†**			
No	46 (30.1%)	52 (34.7%)	98 (32.3%)
Yes	107 (69.9%)	98 (65.3%)	205 (67.7%)

ACPR = Adequate clinical and parasitological response: absence of parasitemia irrespective of fever.

LCF = Late clinical failure: parasitemia beyond day 3 with evidence of fever (clinical malaria).
LPF = Late parasitological failure: parasitemia beyond day 6 without evidence of fever.
†Missing outcomes censored.

Table S3. Repeated measurement of parasite density by microscopy, SBP1, or 18S RNA

	Microscopy (n = 303)			
	**5-Day AL**	**3-Day AL**	**Δ(5D vs. 3D)**	**P-value**
**Overall**	0.718 (0.058)	0.825 (0.058)	-0.107 (0.081)	0.190
**D7**	-0.005 (0.112)	-0.004 (0.112)	0.000 (0.158)	0.998
**D14**	0.011 (0.112)	0.050 (0.112)	-0.040 (0.159)	0.803
**D21**	0.478 (0.112)	0.803 (0.112)	-0.325 (0.158)	0.040
**D28**	1.093 (0.114)	1.169 (0.117)	-0.076 (0.163)	0.640
**D35**	1.258 (0.119)	1.456 (0.120)	-0.198 (0.169)	0.241
**D42**	1.474 (0.124)	1.476 (0.126)	-0.002 (0.177)	0.991

	18S rRNA (n = 273)			
	**5-Day AL**	**3-Day AL**	**Δ(5D vs. 3D)**	**P-value**
**Overall**	2.651 (0.137)	2.823 (0.131)	-0.172 (0.190)	0.366
**D7**	1.661 (0.196)	1.887 (0.186)	-0.227 (0.270)	0.402
**D14**	1.487 (0.196)	1.860 (0.188)	-0.373 (0.272)	0.170
**D21**	2.109 (0.196)	2.815 (0.188)	-0.707 (0.272)	0.009
**D28**	3.225 (0.200)	3.358 (0.195)	-0.133 (0.280)	0.635
**D35**	3.647 (0.205)	3.445 (0.200)	0.202 (0.287)	0.480
**D42**	3.776 (0.213)	3.572 (0.209)	0.204 (0.298)	0.494

	SBP1 mRNA (n = 293)			
	**5-Day AL**	**3-Day AL**	**Δ(5D vs. 3D)**	**P-value**
**Overall**	2.001 (0.124)	2.144 (0.122)	-0.142 (0.174)	0.415
**D7**	0.226 (0.213)	0.284 (0.207)	-0.058 (0.297)	0.845
**D14**	0.348 (0.213)	0.857 (0.209)	-0.510 (0.298)	0.088
**D21**	1.542 (0.213)	2.135 (0.209)	-0.593 (0.298)	0.047
**D28**	2.927 (0.218)	3.179 (0.218)	-0.253 (0.308)	0.413
**D35**	3.328 (0.226)	3.102 (0.224)	0.226 (0.318)	0.477
**D42**	3.637 (0.237)	3.304 (0.236)	0.333 (0.335)	0.320

Least squares mean estimated parasite density comparisons between 3 versus 5-day AL. Standard error of the mean is displayed in parentheses. Models included treatment group, time as categorical variable, an interaction product of treatment group and time, and log_{10} transformed baseline parasite density. The p-values for HIV status and the interaction between HIV status and AL group were not significant; therefore, they were not adjusted for in the final model. P-values are derived from two-sided pairwise contrasts between treatment regimens at each time point.

Table S4. Repeated measurement of mean multiplicity of infection (MOI), and MOI determined by individual sequencing markers

	MOI (n = 285)			
	**5-Day AL**	**3-Day AL**	**Δ(5D vs. 3D)**	**P-value**
**Overall**	5.255 (0.384)	5.519 (0.360)	-0.264 (0.527)	0.617
**D7**	3.598 (1.227)	3.909 (1.145)	-0.311 (1.678)	0.853
**D14**	3.066 (1.157)	5.307 (0.884)	-2.241 (1.457)	0.125
**D21**	5.010 (0.657)	4.915 (0.537)	0.095 (0.848)	0.911
**D28**	7.026 (0.531)	5.957 (0.521)	1.069 (0.744)	0.151
**D35**	6.152 (0.471)	7.125 (0.517)	-0.973 (0.698)	0.164
**D42**	6.680 (0.512)	5.901 (0.536)	0.780 (0.741)	0.294

Repeated measures analysis comparing MOI between 3 versus 5-day AL. Standard error of the mean is displayed in parentheses. Models included treatment group, time as a categorical variable, an interaction product of treatment group and time, and baseline MOI. The p-values for HIV status and the interaction between HIV status and AL regimen were not significant; therefore, they were not adjusted for in the final model. P-values are derived from two-sided interaction terms in the linear mixed-effects model.

Table S5. Children with persistent 18S or SBP1 parasitemia in the early post-treatment period

	18S (%)	SBP1 (%)	Sequenced (%)	Total (n)
**Day 7**	211 (73.8)	22 (7.7)	10 (3.5)	286
HIV-uninfected	159 (74.6)	21 (9.9)	9 (4.2)	213
HIV-infected	52 (71.2)	1 (1.4)	1 (1.4)	73
**Day 14**	184 (65.0)	43 (15.2)	15 (5.3)	283
HIV-uninfected	137 (64.6)	36 (17.0)	13 (6.1)	212
HIV-infected	47 (66.2)	7 (9.9)	2 (2.8)	71

Supplementary Figure 1

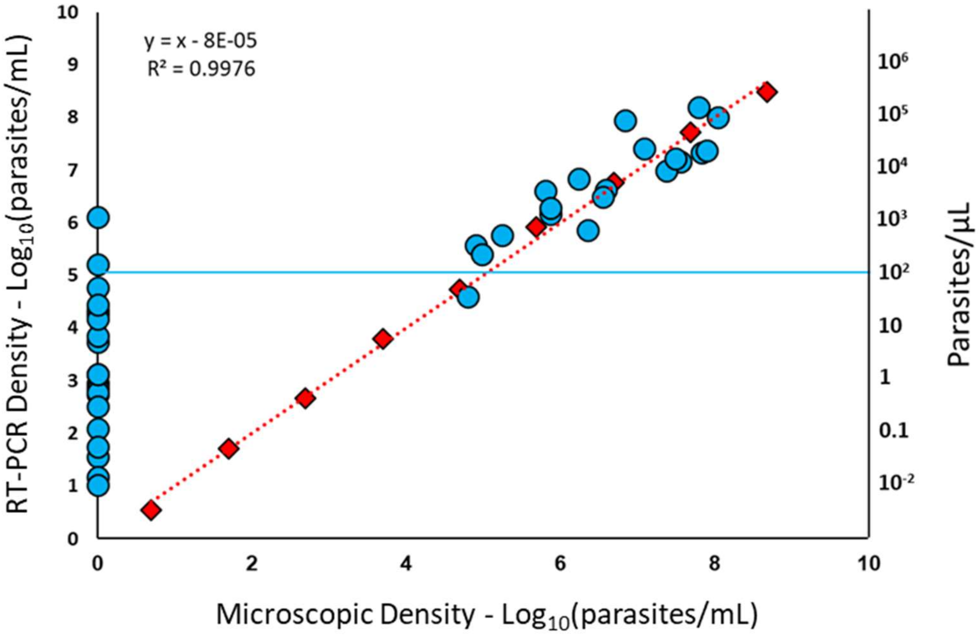

Fig. S1 | Quantification of parasitemia using 18S rRNA. 18S rRNA-determined parasite densities plotted against microscopic parasite densities. Red diamonds and the dotted red line represent a standard curve of known parasite densities performed with every RT-PCR experiment. Blue circles represent experimental samples of HIV-uninfected children (n = 49). The horizontal solid blue line represents the approximate microscopic limit of detection, and the right Y-axis denotes parasites/μL.

Supplementary Figure 2

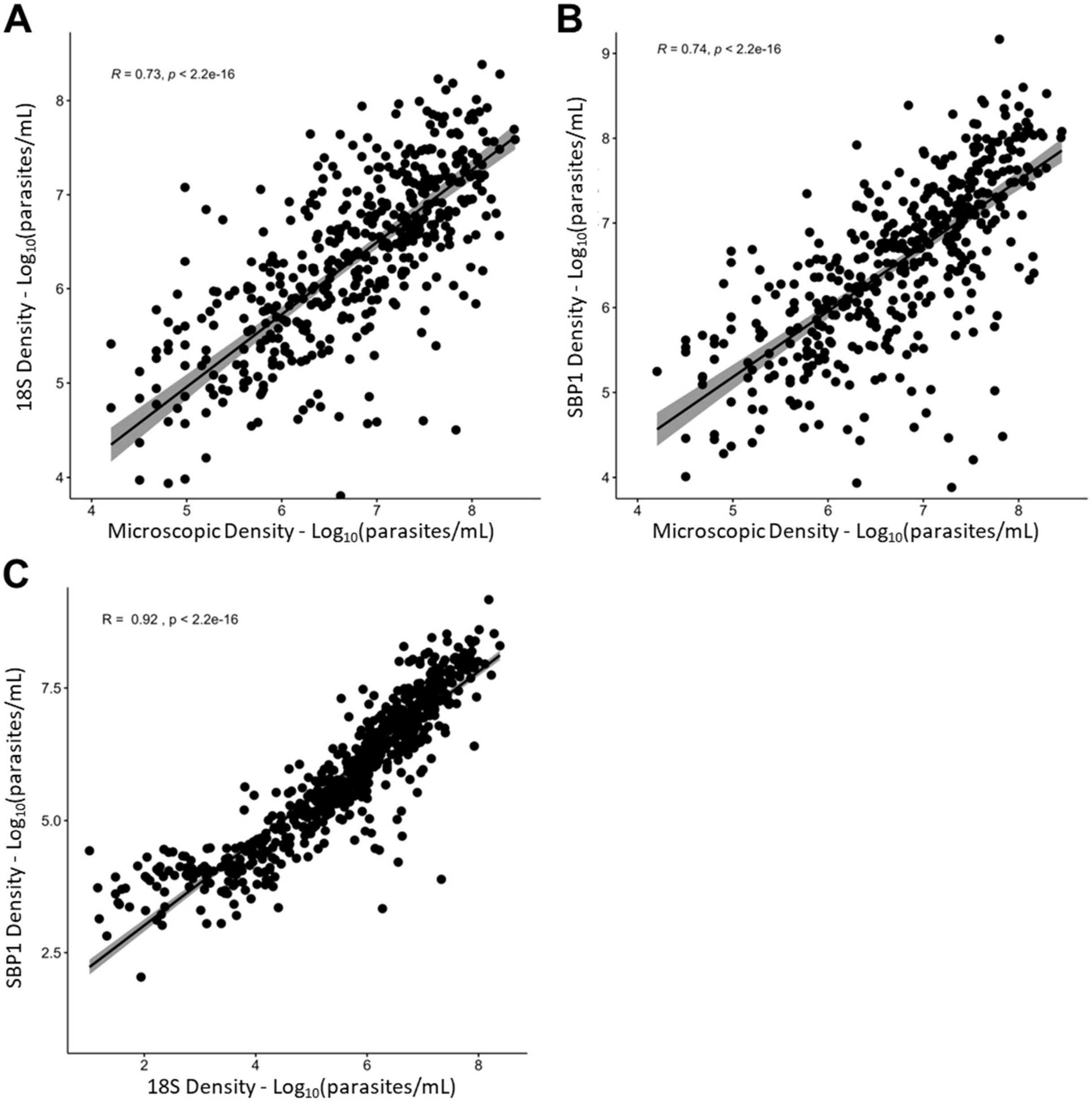

Fig. S2 | Correlation of microscopic, 18S rRNA, and SBP1 mRNA-determined parasite densities. Spearman correlations of microscopic, 18S rRNA, and SBP1 mRNA-determined parasite densities. Solid black line is the best-fit line, and the shaded regions represent the 95% confidence intervals. (**A**) Correlation of 18S parasite densities with microscopic parasite densities (n = 1399). (**B**) Correlation of SBP1 parasite densities with microscopic parasite densities (n =

1379). (**C**) Correlation of 18S parasite densities with SBP1 parasite densities (n = 1387). Densities are linearly correlated until parasitemia drops below 500 parasites/mL, when SBP1 tends to overestimate the parasite density. Limit of detection (LOD) of microscopy = 50,000-100,000 parasites/mL. LOD of 18S rRNA = 5-50 parasites/mL. LOD of SBP1 mRNA = 500-1000 parasites/mL.

Supplementary Figure 3

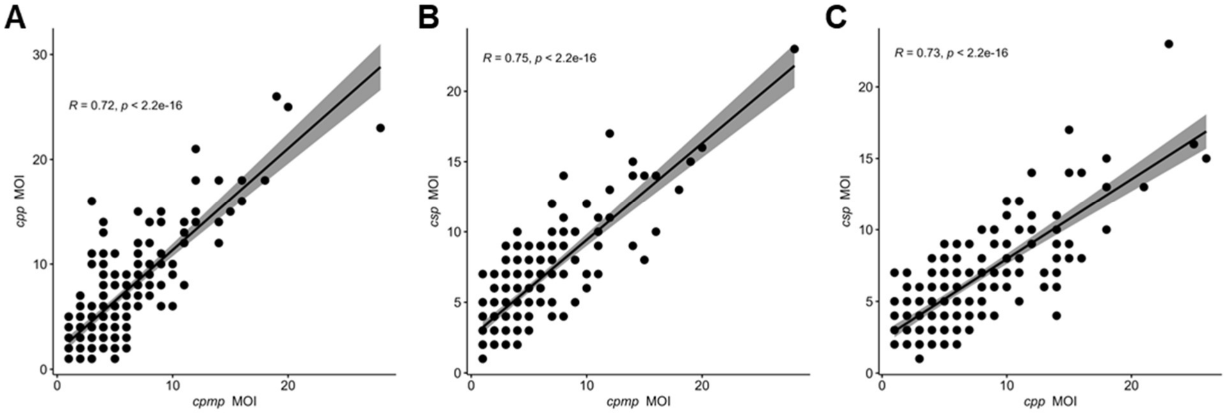

Fig. S3 | Concordance of markers used for amplicon deep sequencing. Spearman correlations of the number of variants identified (defined as multiplicity of infection (MOI)) between *cpmp*, *cpp*, and *csp*. Solid black line is the best-fit line, and the shaded regions represent the 95% confidence intervals. Sample sizes for (**A**) are 192, (**B**) are 193, and (**C**) are 202.

Supplementary Figure 4

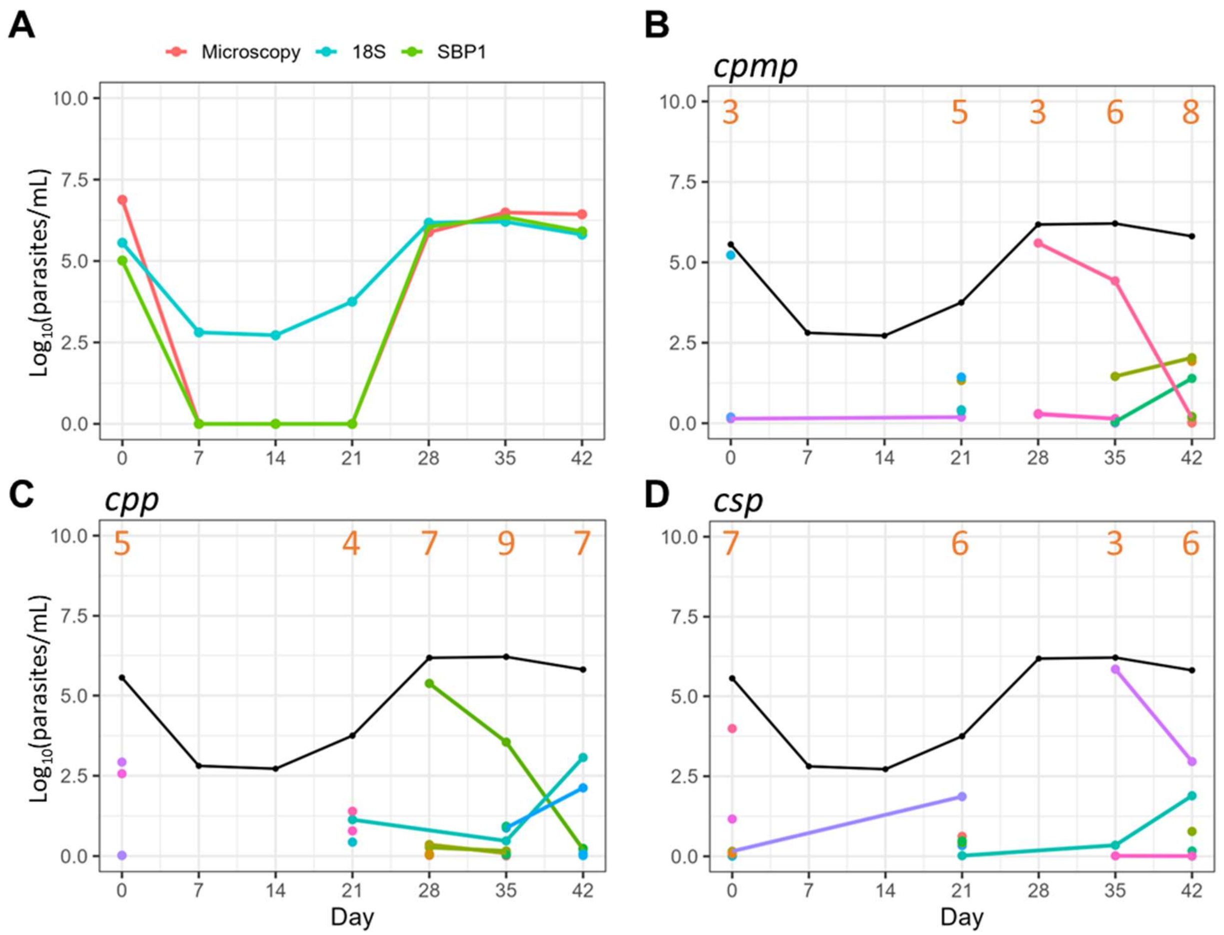

Fig. S4 | Longitudinal parasite densities and clonal dynamics. An example longitudinal curve for a single HIV-uninfected child. (**A**) Longitudinal parasite densities measured using microscopy (red line), 18S rRNA (blue line), and SBP1 (green line). (**B-D**) Longitudinal clonal dynamics measured using amplicon deep sequencing. The solid black line represents 18S rRNA parasite density. The individually colored points represent individual clones with densities determined as a proportion of total reads mapped onto 18S densities. If an individual clone was identified at multiple time points, the points were connected by a matching colored line. The orange numbers along the top of each plot denote the marker-specific MOI for each time point. (**B**) *cpmp* dynamics show a single pre-treatment clone persisted to day 21; new clones appeared on day 21 and subsequent days, with some clones persisting through the end of follow-up. (**C**) *cpp* dynamics

show a similar pattern of new clones appearing on days 21-42, with some clones persisting through the end of follow-up. (**D**) *csp* dynamics confirms the persistence of a pre-treatment clone to day 21; new clones appear on day 21, day 35, and day 42, with some persisting through the end of follow-up (day 28 missing).

Supplementary Figure 5

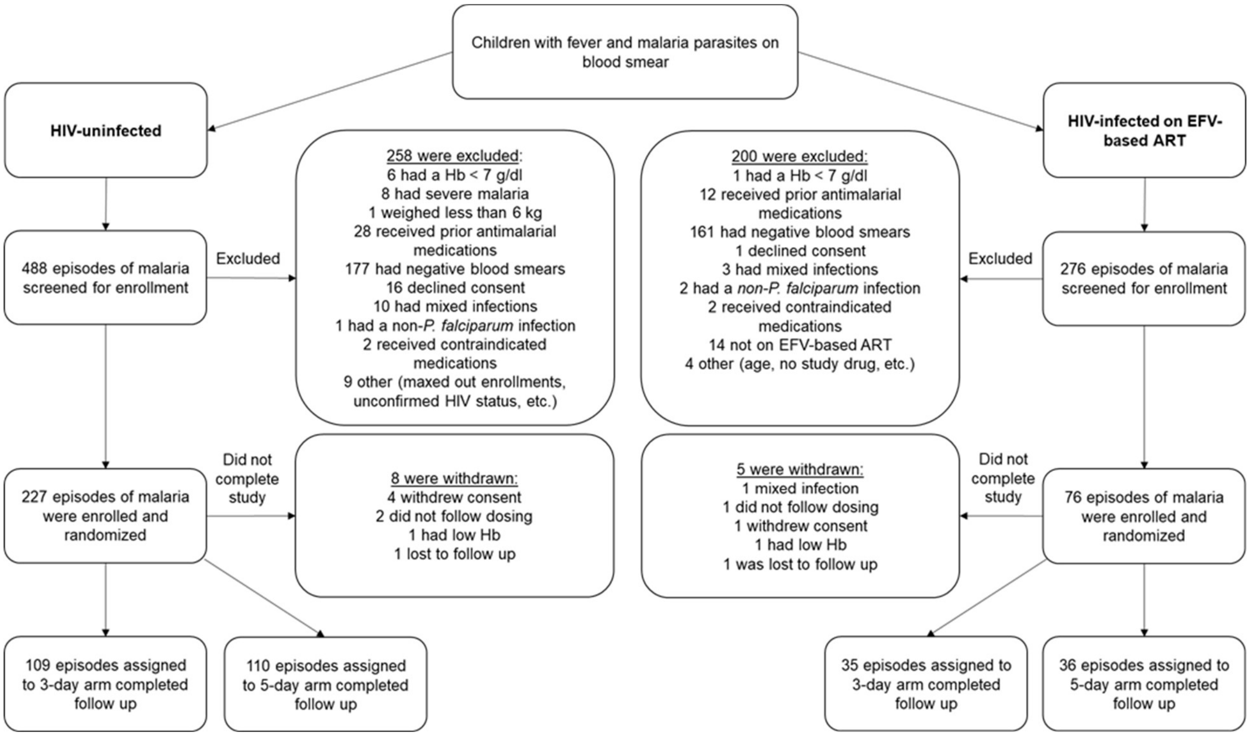

Fig. S5 | CONSORT diagram of EXALT clinical trial. Study screening and enrollment flowchart for the HIV-uninfected and HIV-infected arms showing intention to treat (ITT) and per protocol cohorts. ClinicalTrials.gov number NCT03453840.

Supplementary Methods

RT-PCR primer and probe sequences

Table 1. Primer and probe sequences for *P. falciparum* 18S rRNA and human action

Name	Sequence
Fal TM FOR	5'-CCGACTAGGTGTTGGATGAAAGTGTTAA-3'
Plas TM REV	5'-AACCCAAAGACTTTGATTTCTCATAA -3'
Pf_Ultra_CB	/5Cy5/agcaatctaaaagtcacctcgaaagatgact/3BHQ_2/
Actin_Ultra_F	5'-ACCGAGCGCGGCTACAG-3'
Actin_Ultra_R	5'-CTTAATGTCACGCACGATTTCC-3
Actin_Ultra_P	/VIC/ttcaccaccacggccgagc/MGB/

Table 2. Primer sequences for *P. falciparum* SBP1 mRNA

Name	Sequence
SBP1 FOR	5'-TTGCTAGGTAATATCCTTTTCTTTTTCC-3'
SBP1 REV	5'-GCAAAACAAGCCGTACATGTTG-3'

Amplicon Deep Sequencing primer sequences

1.1 Nest 1 PCR primer sequences

Primer sequences for *P. falciparum* primary PCR targeting *cpmp*

Name	Sequence
cpmp (fw)	CGATACAGGACATATAGA
cpmp (rv)	TTCAATAACATTTACTAGG

Primer sequences for *P. falciparum* primary PCR targeting *cpp*

Name	Sequence
cpp (fw)	TGTCTGAACCAAATTCAA
cpp (rv)	GAATTTGTCACATTTGATGA

Primer sequences for *P. falciparum* primary PCR targeting *csp*

Name	Sequence
csp (fw)	ATCAAGGTAATGGACAAG
csp (rv)	ACTCAAACTAAGATGTGTTC

1.2 Nest 2 PCR Primer sequences

Primer sequences for *P. falciparum* nested PCR targeting *cpmp*

cpmp_fw_linker	GTGACCTATGAACTCAGGAGTCCATAAGTCATTAAAATTTAT GGAT
cpmp_rv_linker	CTGAGACTTGCACATCGCAGCCGTTACTATCAAGATCGTTAATATC

Primer sequences for *P. falciparum* nested PCR targeting *cpp*

Name	Sequence
cpp_fw_linker	GTGACCTATGAACTCAGGAGTCCAAGTTCACTTTTGGGAAATG
cpp_rv_linker	CTGAGACTTGCACATCGCAGCATTACTACCTTTCAGCATATCCGA

Primer sequences for *P. falciparum* nested PCR targeting *csp*

Name	Sequence
csp_fw_linker	GTGACCTATGAACTCAGGAGTCAAATGACCCAAACCGAAATGT
csp_rv_linker	CTGAGACTTGCACATCGCAGCGGAACAAGAAGGATAATACCA

1.3 Adapter sequences

Primer sequences for *P. falciparum* adapter PCR

Forward:
AATGATACGGCGACCACCGAGATCTACACTCTTTCCCTACACGACGCTCTTCCGATCTX
XXXXXXXGTGACCTATGAACTCAGGAGTC

XXXXXXXX = forward barcode

GTGACCTATGAACTCAGGAGTC = linker forward

Reverse:
CAAGCAGAAGACGGCATACGAGATCGGTCTCGGCATTCCTGCTGAACCGCTCTTCCGA
TCTXXXXXXXXCTGAGACTTGCACATCGCAGC

XXXXXXXX = reverse barcode

CTGAGACTTGCACATCGCAGC = linker reverse

1.4 Barcode sequences

Forward barcode for *P. falciparum* adapter PCR forward

Name	Sequence
Fw_2	CTCTCTAT
Fw_3	TATCCTCT
Fw_4	AGAGTAGA
Fw_5	GTAAGGAG
Fw_6	ACTGCATA
Fw_7	AAGGAGTA
Fw_8	CTAAGCCT
Fw_9	CCGAAGTA
Fw_10	GAGCTGAA
Fw_11	GCGAGTAA

Primer sequences for *P. falciparum* adapter PCR reverse

Name	Sequence
Rv_1	TAAGGCGA
Rv_2	CGTACTAG
Rv_3	AGGCAGAA
Rv_4	TCCTGAGC
Rv_5	GGACTCCT
Rv_6	TAGGCATG
Rv_7	CTCTCTAC
Rv_8	CAGAGAGG
Rv_9	GCTACGCT
Rv_10	CGAGGCTG
Rv_11	AAGAGGCA
Rv_12	GTAGAGGA

Supplementary References

1. Beshir KB, Sutherland CJ, Sawa P, et al. Residual Plasmodium falciparum parasitemia in Kenyan children after artemisinin-combination therapy is associated with increased transmission to mosquitoes and parasite recurrence. *J Infect Dis*. Dec 15 2013;208(12):2017-24. doi:10.1093/infdis/jit431
2. Chang HH, Meibalan E, Zelin J, et al. Persistence of Plasmodium falciparum parasitemia after artemisinin combination therapy: evidence from a randomized trial in Uganda. *Sci Rep*. May 20 2016;6:26330. doi:10.1038/srep26330
3. Tadesse FG, Lanke K, Nebie I, et al. Molecular Markers for Sensitive Detection of Plasmodium falciparum Asexual Stage Parasites and their Application in a Malaria Clinical Trial. *Am J Trop Med Hyg*. Jul 2017;97(1):188-198. doi:10.4269/ajtmh.16-0893
4. Oyebola KM, Aina OO, Idowu ET, et al. A barcode of multilocus nuclear DNA identifies genetic relatedness in pre- and post-Artemether/Lumefantrine treated Plasmodium falciparum in Nigeria. *BMC Infect Dis*. Aug 13 2018;18(1):392. doi:10.1186/s12879-018-3314-3
5. Roth JM, Sawa P, Omweri G, et al. Molecular Detection of Residual Parasitemia after Pyronaridine-Artesunate or Artemether-Lumefantrine Treatment of Uncomplicated Plasmodium falciparum Malaria in Kenyan Children. *Am J Trop Med Hyg*. Oct 2018;99(4):970-977. doi:10.4269/ajtmh.18-0233
6. Mwaiswelo R, Ngasala B, Jovel I, et al. Prevalence of and Risk Factors Associated with Polymerase Chain Reaction-Determined Plasmodium falciparum Positivity on Day 3 after Initiation of Artemether-Lumefantrine Treatment for Uncomplicated Malaria in Bagamoyo District, Tanzania. *Am J Trop Med Hyg*. May 2019;100(5):1179-1186. doi:10.4269/ajtmh.18-0729
7. Lubis IND, Wijaya H, Lubis M, et al. Recurrence of Plasmodium malariae and P. falciparum Following Treatment of Uncomplicated Malaria in North Sumatera With Dihydroartemisinin-Piperaquine or Artemether-Lumefantrine. *Open Forum Infect Dis*. May 2020;7(5):ofaa116. doi:10.1093/ofid/ofaa116
8. Mahamar A, Lanke K, Graumans W, et al. Persistence of mRNA indicative of Plasmodium falciparum ring-stage parasites 42 days after artemisinin and non-artemisinin combination therapy in naturally infected Malians. *Malar J*. Jan 9 2021;20(1):34. doi:10.1186/s12936-020-03576-z
9. Beshir KB, Diallo N, Some FA, et al. Persistent Submicroscopic Plasmodium falciparum Parasitemia 72 Hours after Treatment with Artemether-Lumefantrine Predicts 42-Day Treatment Failure in Mali and Burkina Faso. *Antimicrob Agents Chemother*. Jul 16 2021;65(8):e0087321. doi:10.1128/AAC.00873-21
10. Andagalu B, Watson OJ, Onyango I, et al. Malaria Transmission Dynamics in a High-Transmission Setting of Western Kenya and the Inadequate Treatment Response to Artemether-Lumefantrine in an Asymptomatic Population. *Clin Infect Dis*. Feb 18 2023;76(4):704-712. doi:10.1093/cid/ciac527
11. Tadele G, Jaiteh FK, Oboh M, et al. Persistence of Residual Submicroscopic P. falciparum Parasitemia following Treatment of Artemether-Lumefantrine in Ethio-Sudan Border, Western Ethiopia. *Antimicrob Agents Chemother*. Sep 20 2022;66(9):e0000222. doi:10.1128/aac.00002-22
