# Supplementary material for: Persistent and multiclonal malaria parasite dynamics despite extended artemether-lumefantrine treatment in children
Source: Nat Commun. 2024 May 7;15:3817. doi: 10.1038/s41467-024-48210-7 (PMC11076639; doi:10.1038/s41467-024-48210-7)
Supplement: Supplementary file 3 — Reporting Summary [file 41467_2024_48210_MOESM3_ESM.pdf]

nature portfolio

Corresponding author(s): Sunil Parikh

Last updated by author(s): Apr 24, 2024

Reporting Summary

Nature Portfolio wishes to improve the reproducibility of the work that we publish. This form provides structure for consistency and transparency in reporting. For further information on Nature Portfolio policies, see our Editorial Policies and the Editorial Policy Checklist.

Statistics

For all statistical analyses, confirm that the following items are present in the figure legend, table legend, main text, or Methods section.

n/a	Confirmed	
☐	☒	The exact sample size (n) for each experimental group/condition, given as a discrete number and unit of measurement
☐	☒	A statement on whether measurements were taken from distinct samples or whether the same sample was measured repeatedly
☐	☒	The statistical test(s) used AND whether they are one- or two-sided<br>*Only common tests should be described solely by name; describe more complex techniques in the Methods section.*
☐	☒	A description of all covariates tested
☐	☒	A description of any assumptions or corrections, such as tests of normality and adjustment for multiple comparisons
☐	☒	A full description of the statistical parameters including central tendency (e.g. means) or other basic estimates (e.g. regression coefficient) AND variation (e.g. standard deviation) or associated estimates of uncertainty (e.g. confidence intervals)
☐	☒	For null hypothesis testing, the test statistic (e.g. F, t, r) with confidence intervals, effect sizes, degrees of freedom and P value noted<br>*Give P values as exact values whenever suitable.*
☒	☐	For Bayesian analysis, information on the choice of priors and Markov chain Monte Carlo settings
☒	☐	For hierarchical and complex designs, identification of the appropriate level for tests and full reporting of outcomes
☐	☒	Estimates of effect sizes (e.g. Cohen's d, Pearson's r), indicating how they were calculated

Our web collection on statistics for biologists contains articles on many of the points above.

Software and code

Policy information about availability of computer code

Data collection	Clinical trial data was collected using Excel 2016 and Access 2016 (Microsoft Corporation). RT-PCR data was collected using LightCycler® 96 v1.1.0 (Roche Diagnostics International). Amplicon sequencing data was processed using Geneious Prime v2023 (Dotmatics).
Data analysis	Data analysis was performed using R v4.3.0 (R Core Team) and SAS v9.4 M8 (SAS Institute). Amplicon sequencing data was analyzed using DADA2 v1.18 package in R. Relevant code detailing DADA2 analysis has been deposited in Figshare and can be accessed at: https://doi.org/10.6084/m9.figshare.25632636

For manuscripts utilizing custom algorithms or software that are central to the research but not yet described in published literature, software must be made available to editors and reviewers. We strongly encourage code deposition in a community repository (e.g. GitHub). See the Nature Portfolio guidelines for submitting code & software for further information.

Data

Policy information about availability of data

All manuscripts must include a data availability statement. This statement should provide the following information, where applicable:

- Accession codes, unique identifiers, or web links for publicly available datasets
- A description of any restrictions on data availability
- For clinical datasets or third party data, please ensure that the statement adheres to our policy

A data availability statement is included in the manuscript. The relevant data and code that support the findings of this study have been deposited in Figshare and can be accessed at: https://doi.org/10.6084/m9.figshare.25632636. Source data are provided with this paper. All raw sequencing data that support the findings of

this study have been deposited in the NIH NCBI Sequence Read Archive under BioProject accession number PRJNA1034496: https://www.ncbi.nlm.nih.gov/bioproject/PRJNA1034496.

Research involving human participants, their data, or biological material

Policy information about studies with human participants or human data. See also policy information about sex, gender (identity/presentation), and sexual orientation and race, ethnicity and racism.

Reporting on sex and gender	All sampled individuals, including females, were included in this study. There were no exclusion criteria in the original clinical trial based on sex.
Reporting on race, ethnicity, or other socially relevant groupings	Our study group is composed of HIV-uninfected children ages 6 months to 18 years, and HIV-infected children ages 3 to 18 years who presented to the study clinic at Masafu General Hospital in Busia, Uganda. We included all sampled individuals, including all females and all ethnic groups (including ethnic minorities), in the analysis. We did not collect data regarding ethnicity. Parental consent was given for all children enrolled in the study, and child assent was given for all children ages 12-18 years, according to Ugandan requirements.
Population characteristics	HIV-infected and HIV-uninfected Ugandan children between the ages of 6 months to 18 years.
Recruitment	Children will be enrolled from TDH or other referral site or MGH in the Busia District. Parents/guardians of children will be approached for their willingness to participate in intensive PK studies and/or population PK studies for one of more episodes of malaria. All children will undergo 42 days of follow-up around each malaria episode for which they will have PK sampling carried out. Participants may be recruited through multiple referral mechanisms including:<br>1. Tororo District Hospital (TDH). Tororo District Hospital Pediatric, Antenatal and Adult Clinics and Wards: HIV-infected and HIV-uninfected children presenting with uncomplicated malaria to the TDH clinics or wards.<br>2. The AIDS Service Organization (TASO, Tororo). TASO has been providing care to HIV-infected children and adults in Tororo since 1989. HIV-infected children presenting with uncomplicated malaria.<br>3. Masafu General Hospital, Busia District. HIV-infected and HIV-uninfected children presenting with uncomplicated malaria to the clinics or wards.
Ethics oversight	Ethical approval was obtained from the Uganda National Council of Science and Technology, the Makerere University School of Medicine Research Ethics Committee, the University of California, San Francisco Committee on Human Research, and the Yale University Human Investigations Committee.

Note that full information on the approval of the study protocol must also be provided in the manuscript.

Field-specific reporting

Please select the one below that is the best fit for your research. If you are not sure, read the appropriate sections before making your selection.

[x] Life sciences [ ] Behavioural & social sciences [ ] Ecological, evolutionary & environmental sciences

For a reference copy of the document with all sections, see nature.com/documents/nr-reporting-summary-flat.pdf

Life sciences study design

All studies must disclose on these points even when the disclosure is negative.

Sample size	This study included 303 children. Sample size was determined based on detecting differenecs in pharmacokinetic parameters. Please refer to Whalen et. al. Clin Infect Dis. 2023 Feb 8;76(3):443-452. doi: 10.1093/cid/ciac783. PMID: 36130191 for a full description of sample size analysis.
Data exclusions	For RT-PCR, no data was excluded, but any Ct value > 37 was interpreted as negative. For amplicon sequencing, samples with < 100 reads or variants with a within-host haplotype frequency < 0.1% were excluded from the analysis. For statistical analysis, any subjects with missing outcomes were censored.
Replication	For RT-PCR, all samples were analyzed as singlicate. Standard curves were run in duplicate. For amplicon sequencing, 40 samples were analyzed as technical duplicates to validate reproducibility (data not included in the manuscript). These attempts at replication were successful. The remaining sequencing samples were analyzed as singlicate. Samples were analyzed as singlicate based on study objectives and the number of samples involved (to define longitudinal clonal dynamics over a 6 week period across a large number of study participants with over 2,000 samples across three sequencing markers).
Randomization	Children were randomized using a computer-generated randomization list. Allocation sequence was concealed from the clinician assessing and enrolling participants through sequentially numbered, opaque, sealed envelopes assembled by a third-party not involved in participant enrollment, treatment, or follow-up.
Blinding	This study was open-label with weight-based artemether-lumefantrine dosing.

Reporting for specific materials, systems and methods

We require information from authors about some types of materials, experimental systems and methods used in many studies. Here, indicate whether each material, system or method listed is relevant to your study. If you are not sure if a list item applies to your research, read the appropriate section before selecting a response.

Materials & experimental systems

n/a	Involved in the study
x	Antibodies
	x Eukaryotic cell lines
x	Palaeontology and archaeology
x	Animals and other organisms
	x Clinical data
x	Dual use research of concern
x	Plants

Methods

n/a	Involved in the study
x	ChIP-seq
x	Flow cytometry
x	MRI-based neuroimaging

Eukaryotic cell lines

Policy information about cell lines and Sex and Gender in Research

Cell line source(s)	Plasmodium falciparum strains 3D7 and Dd2; Malaria Research and Reference Reagent Resource Center (BEI Resources, NIAID, NIH).
Authentication	All cell lines were authenticated by ATCC on behalf of BEI Resources and provided with a certificate of authentication (see https://www.beiresources.org/Catalog/BEIParasiticProtozoa/MRA-102.aspx).
Mycoplasma contamination	All cell lines tested negative for mycoplasma.
Commonly misidentified lines (See ICLAC register)	No commonly misidentified cell lines were used.

Clinical data

Policy information about clinical studies
All manuscripts should comply with the ICMJEguidelines for publication of clinical research and a completedCONSORT checklist must be included with all submissions.

Clinical trial registration	ClinicalTrials.gov number NCT03453840
Study protocol	The full study protocol can be found on ClinicalTrials.gov at: https://storage.googleapis.com/ctgov2-large-docs/40/NCT03453840/Prot_SAP_002.pdf
Data collection	Clinical trial data collection was performed between Feb 19, 2018 to Aug 31, 2021. The study was conducted at the Masafu General Hospital (0.41310 N, 34.03502 E) located in rural Busia, Uganda. The study clinic is equipped with a patient care and triage center, pharmacy, and a laboratory with a microscopy facility.
Outcomes	Primary outcomes were (1) comparative plasma PK parameters for artemether, DHA, and lumefantrine between regimens and (2) microscopy determined recurrent parasitemia. Secondary outcomes were genotype-unadjusted/adjusted recurrent malaria at 28 and 42 days using standard WHO criteria. For the primary PK outcome, drug concentrations were assessed using validated liquid chromatography tandem mass spectrometry. For primary and secondary parasitological outcomes, parasitemia was assessed using a combination of active and passive surveillance. PCR-corrected treatment failure was performed using size polymorphism-based genotyping and capillary electrophoresis for msp2, msp1, and 4 microsatellites.

Plants

Seed stocks	*Report on the source of all seed stocks or other plant material used. If applicable, state the seed stock centre and catalogue number. If plant specimens were collected from the field, describe the collection location, date and sampling procedures.*
Novel plant genotypes	*Describe the methods by which all novel plant genotypes were produced. This includes those generated by transgenic approaches, gene editing, chemical/radiation-based mutagenesis and hybridization. For transgenic lines, describe the transformation method, the number of independent lines analyzed and the generation upon which experiments were performed. For gene-edited lines, describe the editor used, the endogenous sequence targeted for editing, the targeting guide RNA sequence (if applicable) and how the editor was applied.*
Authentication	*Describe any authentication procedures for each seed stock used or novel genotype generated. Describe any experiments used to assess the effect of a mutation and, where applicable, how potential secondary effects (e.g. second site T-DNA insertions, mosiacism, off-target gene editing) were examined.*
